# Supplementary material for: Toxoplasma gondii Rhoptry Protein 7 (ROP7) Interacts with NLRP3 and Promotes Inflammasome Hyperactivation in THP-1-Derived Macrophages
Source: Cells. 2022 May 12;11(10):1630. doi: 10.3390/cells11101630 (PMC9139738; doi:10.3390/cells11101630)
Supplement: Supplementary file 1 [file cells-11-01630-s001.zip › Supplemental Figures.pdf]

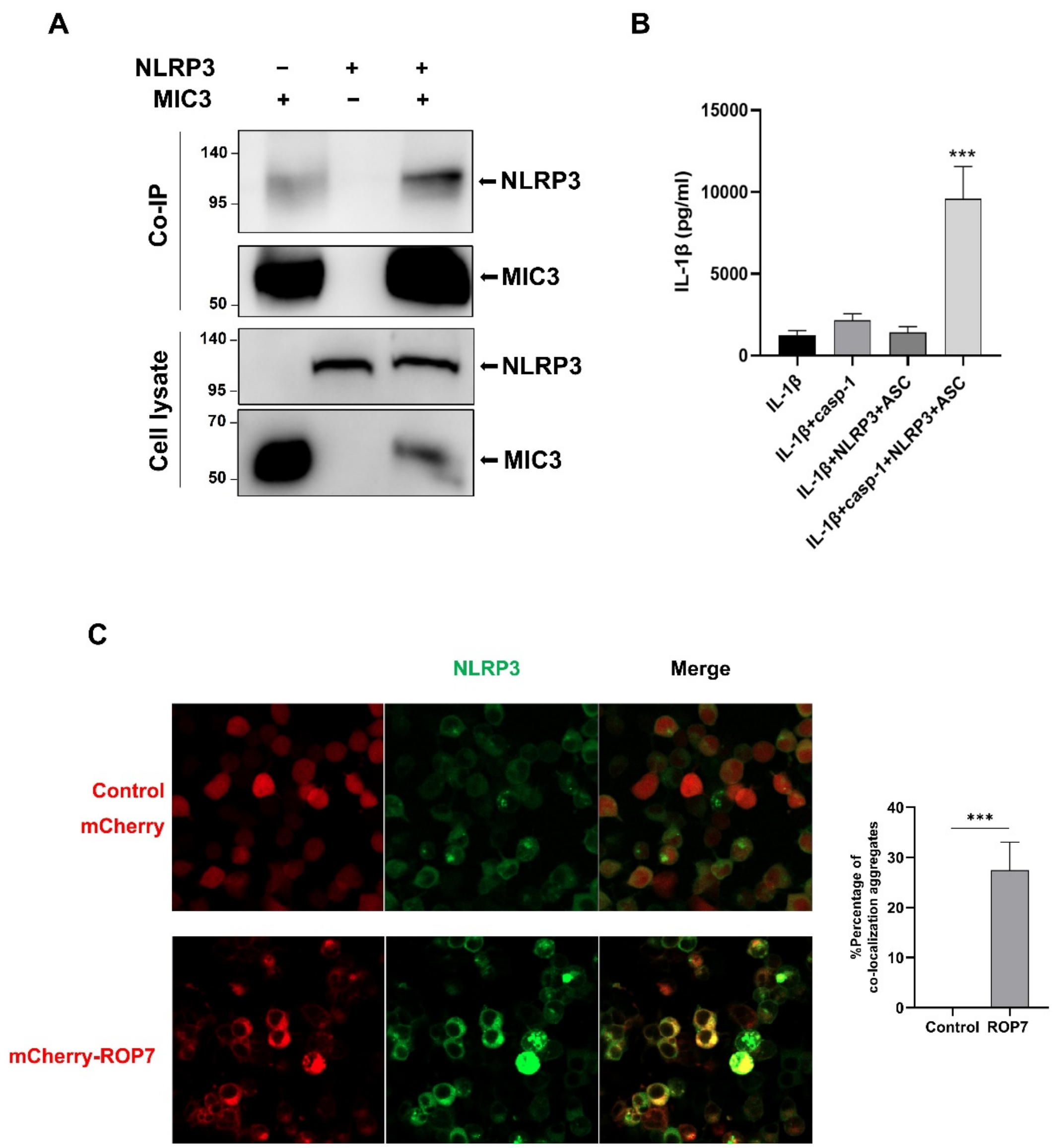

Figure S1. (**A**) The 293T cells were transfected with pcDNA3-HA-MIC3 and pcDNA3.1-Flag-NLRP3. The cell lysates were subjected to immunoprecipitation using anti-HA antibody and further analyzed by immunoblotting with an anti-Flag antibody (NLRP3) and an anti-HA antibody (MIC3). (**B**) The 293T cells were co-transfected with ROP7 and indicated inflammasome-component plasmids. The IL-1β secretion in cell-free supernatant was measured after 36 h. Data are expressed as mean ± SEM values. ***$p < 0.001$. (**C**) The 293T cells were transfected with pcDNA3-NLRP3-EGFP and pmCherry or pmCherry-ROP7. At 48 h post-infection, ROP7 and NLRP3 co-localization was captured via confocal microscopy. The number of co-localization aggregates was counted through multiple fields of microscope from three independent experiments. Data were expressed as mean percentage ± SEM values. **$p < 0.01$

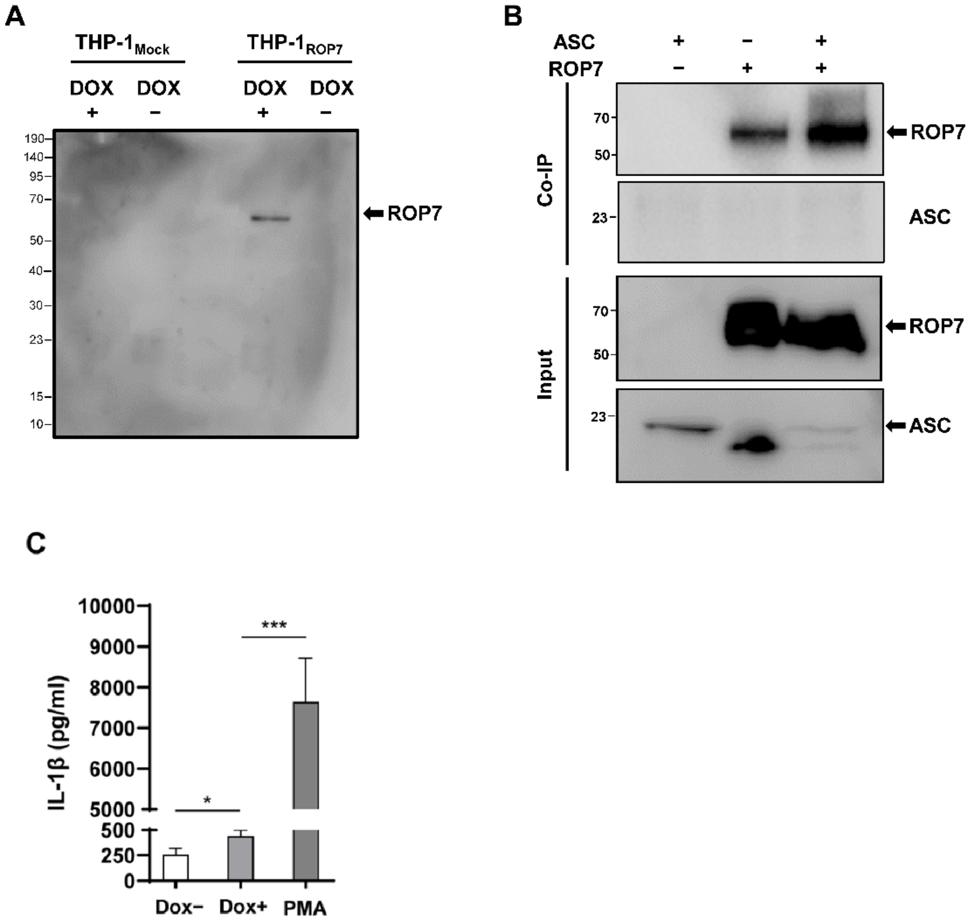

Figure S2. (**A**) THP-1 expressed Flag-HA-ROP7 in presence of doxycycline. The THP-1 cells were induced by 1 μg doxycycline for 48 h and differentiated under 100 μM PMA for another 48 h. Cells were harvested for Western blot using anti-HA. (**B**) The 293T cells were transfected with pcDNA3-HA-ROP7 and pcDNA3.1-Flag-ASC. The cell lysates were subjected to immunoprecipitation using anti-HA antibody and further analyzed by immunoblotting with an anti-Flag antibody (ASC) and an anti-HA antibody (ROP7). (**C**) IL-1β secreted continuously in macrophages but not in monocytes. THP-1$_{ROP7}$ were induced by 1μg/ml doxycycline or not for 48 h, and the doxycycline-induced one were further differentiated under 100 nM PMA for another 48 h. The supernatants were collected for ELISA to detect IL-1β. Data are expressed as mean ± SEM values. *$p < 0.05$, ***$p < 0.001$.

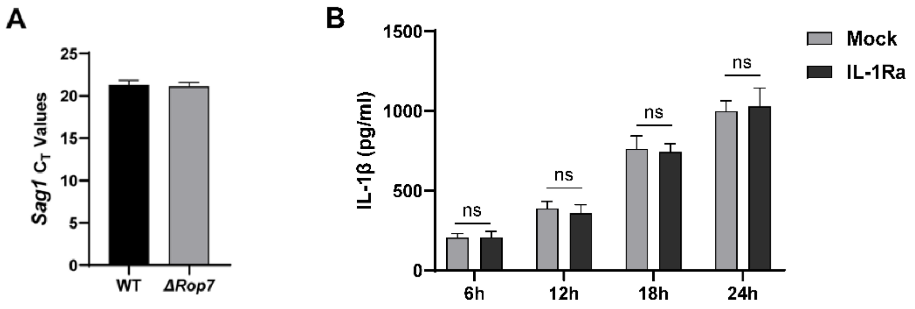

Figure S3. (**A**) THP-1 were differentiated under 100-nM PMA for 48 h. After PMA differentiation, the THP-1 cells were infected with WT or Δ*Rop7* tachyzoites at MOI = 1. The cells were harvest to detect approximate quantity of tachyzoite *via* qPCR according to *Sag1* transcription (C_T values). (**B**) THP-1 were differentiated under 100-nM PMA for 48 h and treated with 30-μM IL-1Ra or nothing for 1 h before parasite invasion. The cells were infected with parasites (MOI = 1) with 30-μM IL-1Ra or none. The IL-1β in supernatants was detected by ELISA. Data are expressed as mean ± SEM values.
